# A Case of Successfully Treated Central Line-Associated Bloodstream Infection Due to Vancomycin-Resistant Leuconostoc Citreum in a Child With Biliary Atresia

**DOI:** 10.7759/cureus.21227

**Published:** 2022-01-14

**Authors:** Ali Modaweb, Zahraa Mansoor, Ali Alsarhan, Walid Abuhammour

**Affiliations:** 1 Pediatrics, Al Jalila Children's Speciality Hospital, Dubai, ARE; 2 General Pediatrics, Al Jalila Children's Speciality Hospital, Dubai, ARE; 3 Infectious Diseases, Al Jalila Children's Speciality Hospital, Dubai, ARE

**Keywords:** biliary atresia, vancomycin-resistant bacteria, leuconostoc citreum, central line-associated infections (clabsi), central venous catheter (cvc)

## Abstract

Infections caused by the Leuconostocspecies have been increasingly described in the literature. They are Gram-positive, catalase-negative cocci that are intrinsically resistant to glycopeptides, including vancomycin. Although rarely pathogenic in humans, they have been primarily found in patients with immunosuppression, and a history of prolonged antibiotics use. We report a rare case of central venous catheter (CVC) infection caused by *Leuconostoc citreum*, which was successfully treated with salvaging of the CVC, aiming to highlight the potential risk factors and share the course of management of our patient.

## Introduction

Central venous catheter (CVC) has an essential role in the management of patients with chronic medical conditions who require complex care. CVC’s are frequently used for regular medication administration, blood sampling, and delivery of specialized treatment such as hemodialysis and parenteral nutrition. Despite their utility, these devices predispose patients to various complications including the risk of central line-associated bloodstream infection (CLABSI) [1,2].

Typically, tunneled catheters are less susceptible to CLABSI because they have a cuff that causes a fibrotic reaction around the catheter, creating a barrier that prevents bacterial migration [1]. On the contrary, non-tunneled catheters are more susceptible to CLABSI due to the absence of the tunnel (a subcutaneous tract), and this risk is further potentiated by the long duration of indwelling, extensive replacement, frequent repositioning, and poor handling [1,3].

CLABSI is most commonly caused by Gram-positive organisms including coagulase-negative Staphylococcus spp. (37.8%), Enterococcus (11.2%), and *Staphylococcus aureus* (9.3%), and is considered a major cause of prolonged hospitalization and increased mortality [2,3]. *Leuconostoc citreum* are Gram-positive, catalase-negative cocci, that belong to the family Lactobacillaceae [4]. They are primarily known to be resistant to glycopeptides including vancomycin [5]. The Leuconostoc species have been recently identified to cause serious infections, including bacteremia, urinary tract infections, pneumonia, meningitis, endocarditis, liver and lung abscesses, peritonitis, and intra-abdominal infections [6-13]. Reported cases of infections caused by the Leuconostoc species are limited in the literature. Most of these cases occurred in patients who are immunosuppressed or have previously received prolonged courses of antibiotics [5,14]. In pediatrics, they were more common in premature children and those born with low birth weight [14].

We report a rare case of CVC infection caused by *Leuconostoc citreum*, in a CVC-dependent complex need child, with the aim of highlighting the risk factors that predisposed our patient to this infection and sharing our experience in the management of this rare bacteria. The infection was treated with intravenous (IV) ampicillin, in addition to salvaging the line.

## Case presentation

A 17-month-old male, an ex-preterm baby (34 weeks) with low birth weight (2.2 kg), who is a known case of biliary atresia post failed Kasai procedure, failure to thrive, and renal tubulopathy was admitted to our hospital for nutrition and treatment. He has been admitted for over one year after initially presenting with ascending cholangitis. Due to his complex medical background, the child had a tunneled Broviac catheter (Salt Lake, UT: Bard Access Systems, Inc.) by which he received total parenteral nutrition (TPN), along with frequent corrections of electrolyte imbalances given his renal disorder. The child also had a history of recurrent infections during his hospital stay including ascending cholangitis, urinary tract infection, pneumonia, which necessitated prolonged courses of antibiotics. The most recent infection he acquired was a *Klebsiella pneumoniae* CLABSI infection for which his CVC was replaced two weeks prior. The antibiotics he received included penicillin, β-lactam, cephalosporins, and glycopeptides including vancomycin.

The child developed a persistent low-grade fever with no other symptoms or focus of infection. He subsequently underwent septic workup including central line cultures to evaluate for CLABSI. His blood tests showed white blood cells (WBC) of 14.5 x 10^9^/L (normal range: 5-15 x 10^9^/L) and an absolute neutrophil count (ANC) of 500/μL (normal range: 1500-8000/μL). C-reactive protein (CRP) was 0.89 mg/L (normal range: 0.0-2.8 mg/L) and procalcitonin (PCT) was 0.25 ng/mL (normal range: 0.0-0.5 ng/mL). His liver enzymes were at baseline (Table 1). A respiratory viral polymerase chain reaction (PCR) panel and coronavirus disease 2019 (COVID-19) PCR from nasopharyngeal aspirate were negative. On day one, the blood culture obtained from the central catheter was flagged for positive growth in BactAlert (Marcy-l'Étoile, France: BioMérieux), and the Gram stain revealed Gram-positive cocci in pairs and in chains. On day two, small, smooth, round <1 mm, and grayish colonies were identified on blood agar. No colony growth was observed on MacConkey agar. Catalase test was negative. On day four, *Leuconostoc citreum* was identified with 99% probability using the VITEK 2 (Marcy-l'Étoile, France: BioMérieux) Gram-positive identification card (BioMérieux). Antibiotic susceptibility was tested using Clinical and Laboratory Standards Institute (CLSI) M45 recommendation of media and antibiotic breakpoints. This organism is intrinsically resistant to vancomycin, hence routine testing for susceptibility was not done. The organism was sensitive to penicillin and ampicillin. Minimum inhibitory concentration (MIC) breakpoints for ceftriaxone and clindamycin were not available in CLSI. Three subsequent central blood cultures continued to grow the same microorganism. Blood cultures obtained from the peripheral samples and urine culture were negative. Echocardiogram did not reveal any vegetation.

**Table 1:** Summary of basic laboratory investigations.

Investigations		Result	Normal Range
Compete blood count	Hemoglobin (Hb)	7.4g/dL	11-14g/dL
	White cell count	14.5 × 10^9^/L	5-15 × 10^9^/L
	Absolute neutrophil count	500/μL	1500-8000/μL
	Platelet count	141 × 10^9^/L	200-490 × 10^9^/L
Inflammatory markers	C-reactive protein (CRP)	0.89 mg/L	0.0-2.8 mg/L
	Procalcitonin (PCT)	0.25 ng/mL	0.0-0.5 ng/mL
Liver function tests	Aspartate aminotransferase (AST)	42 U/L	0-110 U/L
	Alanine aminotransferase (ALT)	22 U/L	0-39 U/L
	Alkaline phosphatase	4029 IU/L	0-281 IU/L
	Albumin	2.7 g/dL	3.8-5.4 g/dL
	Total bilirubin	2.18 mg/dL	0.00-1.20 g/dL
Renal function tests	Urea	38 mg/dL	11-36 mg/dL
	Creatinine	0.30 mg/dL	0.00-0.41 mg/dL
Electrolytes	Sodium	148 mmol/L	136-145 mmol/L
	Potassium	3.8 mmol/L	3.4-5.1 mmol/L
	Calcium	7.7 mg/dL	9-11 mg/dL

He was started empirically on vancomycin and ceftriaxone which he received for four days, taking into consideration his previous *Klebsiella pneumoniae* CLABSI infection. However, he continued to spike low-grade fevers despite being on antibiotics. He was switched, on day four, to a single daily dose of IV ampicillin 200mg/kg/day, once *Leuconostoc citreum* was identified in his blood culture. It was then continued for 10 days from the first negative blood culture, and a total duration of 12 days. He remained afebrile for four days prior to discontinuing ampicillin, and the CVC was salvaged.

## Discussion

*Leconostoc citreum* is currently one of 10 species assigned to the family Lactobacillaceae, including (*Leuconostoc mesenteroides*, *Lactococcus lactis*) [4]. They are known to be used in the industrial fermentation of dairy products and are occasionally found in the human vagina and stool samples [9,15]. They are considered nutritionally fastidious microorganisms; hence have to be cultivated under special circumstances [15].

In the literature, the first case of Leuconostoc was reported in 1985 in France and was initially misreported to be caused by *Streptococcus sanguis* [5]. Its identity was questioned and was later identified as a Leuconostocs species. Since then, it has been further confused with Streptococcus, Enterococcus, and Lactobacillus, and has been mistakenly implicated in other serious infections. CLABSI due to *Leuconostoc mesenteroides* and *Lactococcus lactis* has been described previously in the literature, but not due to *Leconostoc citreum* [8,16]. They are also more common in patients with immunosuppression and have been reported in patients with hematological malignancies, chronic liver failure, and chronic renal disease [9,12,13]. Prolonged use of antibiotics, including β-lactam and vancomycin, also increased the risk of infection with these bacteria.

Although Leuconostoc species have a low pathogenic potential in humans, this rare presentation could be attributed to multiple predisposing risk factors present in our patient [16]. His severe malnutrition, immunosuppression status evident by neutropenia, along with the presence of an indwelling CVC that was only recently replaced, were all contributing factors that increased his risk of contracting a CLABSI. Kelly et al. in 2013 has previously described that neutropenia, defined as an ANC <500 cells/μL in the prior week, was associated with CLABSI among pediatric oncology patients [17]. Furthermore, in a woman that developed neutropenia associated with chemotherapy for acute lymphoblastic leukemia, Ino et al. also found an infection that was confirmed to be *Leuconostoc pseudomesenteroides* [9].

Additionally, malnutrition which is often noted in most children less than two years with biliary atresia was also a significant risk factor in our patient [18]. Triggs et al. in 2019 found that there was a substantial risk for CLABSI among children with biliary atresia listed for liver transplantation, but no independent risk factors were associated with it [18]. Moreover, TPN is considered as an independent risk factor of CLABSI, providing a niche for nutritionally fastidious bacteria [19]. In a retrospective study done by Florescu et al. in 2008 among patients with short bowel syndrome, all patients diagnosed with Leuconostoc bacteremia were receiving TPN by a CVC [20]. Infection with Leuconostoc species has also been found in cases with altered intestinal mucosa, and exposure to intraabdominal surgery.

Handwerger et al. in 1990 reviewed a total of 17 cases due to Leuconostoc species, almost all cases had received previous antibiotic therapy. It was found that using broad-spectrum antibiotics predisposed patients to more frequent isolation of previously rare organisms such as methicillin-resistant *Staphylococcal aureus*. Similarly, the increased use of vancomycin could predispose to increased isolation of vancomycin-resistant microorganisms, including Leuconostoc species [5]. Because no standard susceptibility methods are currently established for *Leuconostoc citreum*, the appropriate treatment remains to be unknown. Therefore, the treatment regimen was guided by the MIC [8]. Nevertheless, previously successful treatment regimens included penicillin G, ampicillin, clindamycin, aminoglycosides, and glycylcyclines [5,6]. For our patient, we opted to use ampicillin due to its bactericidal properties.

## Conclusions

In conclusion, we presented a rare case of CLABSI caused by *Leuconostoc citreum*. This case indicated that infection with Leuconostoc species should be suspected in the setting of CVC-related bacteremia not responding to vancomycin, immunosuppression, and in patients with a history of prolonged antibiotics use. Other high potential risk factors included TPN use and extensive handling of the line, hence highlighting the importance of periodic audits, and following standard guidelines on the care of TPN and central lines to prevent infection. Moreover, until appropriate treatment guidelines are established for this presentation, we suggest using the MIC to guide the treatment regimen.
